# An Interactive Computer Session to Initiate Physical Activity in Sedentary Cardiac Patients: Randomized Controlled Trial

**DOI:** 10.2196/jmir.3759

**Published:** 2015-08-24

**Authors:** Fabio A Almeida, Renae L Smith-Ray, David A Dzewaltowski, Russell E Glasgow, Rebecca E Lee, Deborah SK Thomas, Stanley Xu, Paul A Estabrooks

**Affiliations:** ^1^Implementation and Systems Science LaboratoryDepartment of Human, Nutrition, Foods, and ExerciseVirginia TechRoanoke, VAUnited States; ^2^Center for Research on Health and AgingInstitute for Health Research and PolicyUniversity of Illinois at ChicagoChicago, ILUnited States; ^3^Department of KinesiologyKansas State UniversityManhattan, KSUnited States; ^4^Department of Family MedicineUniversity of Colorado School of MedicineAurora, COUnited States; ^5^College of Nursing and Health InnovationArizona State UniversityTempe, AZUnited States; ^6^Department of Geography and Environmental SciencesUniversity of Colorado DenverDenver, COUnited States; ^7^Institute for Health ResearchKaiser Permanente ColoradoAurora, COUnited States; ^8^Department of Human Nutrition, Foods and ExerciseVirginia Tech and Carilion ClinicRoanoke, VAUnited States

**Keywords:** exercise, physical, treadmill test, human computer interaction, behavioral research, cardiovascular diseases, interactive media

## Abstract

**Background:**

Physical activity (PA) improves many facets of health. Despite this, the majority of American adults are insufficiently active. Adults who visit a physician complaining of chest pain and related cardiovascular symptoms are often referred for further testing. However, when this testing does not reveal an underlying disease or pathology, patients typically receive no additional standard care services. A PA intervention delivered within the clinic setting may be an effective strategy for improving the health of this population at a time when they may be motivated to take preventive action.

**Objective:**

Our aim was to determine the effectiveness of a tailored, computer-based, interactive personal action planning session to initiate PA among a group of sedentary cardiac patients following exercise treadmill testing (ETT).

**Methods:**

This study was part of a larger 2x2 randomized controlled trial to determine the impact of environmental and social-cognitive intervention approaches on the initiation and maintenance of weekly PA for patients post ETT. Participants who were referred to an ETT center but had a negative-test (ie, stress tests results indicated no apparent cardiac issues) were randomized to one of four treatment arms: (1) increased environmental accessibility to PA resources via the provision of a free voucher to a fitness facility in close proximity to their home or workplace (ENV), (2) a tailored social cognitive intervention (SC) using a “5 As”-based (ask, advise, assess, assist, and arrange) personal action planning tool, (3) combined intervention of both ENV and SC approaches (COMBO), or (4) a matched contact nutrition control (CON). Each intervention was delivered using a computer-based interactive session. A general linear model for repeated measures was conducted with change in PA behavior from baseline to 1-month post interactive computer session as the primary outcome.

**Results:**

Sedentary participants (n=452; 34.7% participation rate) without a gym membership (mean age 58.57 years; 59% female, 78% white, 12% black, 11% Hispanic) completed a baseline assessment and an interactive computer session. PA increased across the study sample (*F*_1,441_=30.03, *P<*.001). However, a time by condition interaction (*F*_3,441_=8.33, *P<*.001) followed by post hoc analyses indicated that SC participants exhibited a significant increase in weekly PA participation (mean 45.1, SD 10.2) compared to CON (mean -2.5, SD 10.8, *P*=.004) and ENV (mean 8.3, SD 8.1, *P*<.05). Additionally, COMBO participants exhibited a significant increase in weekly PA participation (mean 53.4, SD 8.9) compared to CON (*P*<.001) and ENV (*P*=.003) participants. There were no significant differences between ENV and CON or between SC and COMBO.

**Conclusions:**

A brief, computer-based, interactive personal action planning session may be an effective tool to initiate PA within a health care setting, in particular as part of the ETT system.

**Trial Registration:**

Clinicaltrials.gov NCT00432133, http://clinicaltrials.gov/ct2/show/NCT00432133 (Archived by WebCite at http://www.webcitation.org/6aa8X3mw1).

## Introduction

Physical inactivity is a known contributing factor to many negative health conditions including cardiovascular disease, obesity, cancer, and diabetes [1,2]. Regular physical activity (PA) leads to improved exercise capacity, improved heart rate, and reduces the risk of cardiovascular disease and mortality [3,4]. Despite this, less than one third of adults in the United States engage in a sufficient amount of leisure time PA according to the Healthy People 2020 guidelines [5]. Regular PA is recommended by the American College of Sport Medicine and the American Heart Association (AHA) as tertiary prevention for older adults who have been diagnosed with coronary heart disease (CHD) and hypertension [4,6].

As a result, both individual-level and environmental-level strategies have been used to promote PA behavior change [7-10]. Many of these strategies have used social cognitive theory as a theoretical basis to help predict and improve individual-level PA participation [7,11]. On the other hand, social ecological theories of PA claim that individual-level factors alone are not sufficient predictors of PA participation; instead, environmental accessibility also plays an important role [9,10]. While it is clear that different theoretical underpinnings help predict PA participation, it is unclear whether individual or environmental factors have a greater impact on short-term PA outcomes.

In addition to having a strong theoretical basis, the intervention setting also plays an important part on PA behavior change. A review of 73 studies commissioned by the US Preventive Task Force (USPTF) concluded that behavioral counseling by physicians and other health facility-based practitioners that includes PA is associated with improving indicators of cardiac health [12]. However, studies show that both patients and physicians reported that less than 50% of patients ever receive any PA counseling as part of their regular office visits [13-16]. This could be due to competing demands, lack of PA training and or knowledge by physicians, or time constraints within a busy health care practice. For these reasons, the feasibility of incorporating a PA intervention into the health care delivery system is challenging. Furthermore, a Cochrane review showed that tailored rather than general PA information is more likely to help patients engage in PA, but that tailored PA information coupled with information on outside exercise resources was more effective than brief physician counseling on PA alone [17].

Limited information is available on feasible approaches for delivering tailored, clinic-based PA counseling that includes resources for PA outside of the health care system. In Australia, Eakin et al have demonstrated that PA can be increased when health care settings use telephone-counseling approaches [18]. In the United States, the Activity Counseling Trial [19] used a combination of physician advice and onsite health educators with the telephone support to successfully improve cardiorespiratory fitness among women with no improvement reported among men. However, these approaches have not been translated to clinical settings in the United States and are still resource intensive for delivery across a population of patients.

On the other hand, technology-based approaches have the potential to enhance feasibility and dissemination of this type of clinic-based PA intervention [20]. Technology-based interventions allow for rapid dissemination of health messages using one or more forms such as computer/Internet, smart phone apps, text messaging, and interactive voice response [21,22]. Technology-based interventions are a resource-efficient and affordable delivery method that enables individual tailoring and adaptation, frequent assessment, automated treatment delivery, and the potential to reach large samples [21-24]. Interactive computer sessions have been used to target nutrition and PA knowledge, dietary patterns, PA participation, and exercise self-efficacy in various settings [25-29].

Early evidence for the effectiveness of tailored technology-based PA programs was mixed [30-32], but more recent research suggests that eHealth interventions have a significant positive impact on PA participation and quality of life, and they improve maintenance of health behavior change [22,29,33-35]. Many eHealth studies have faced methodological challenges including accessibility of mobile devices such as smartphones, high attrition, and self-reported measurement [22,30,31,34]. However, Mounton and Cloes found that, compared to a Web-based intervention, a center-based PA intervention was superior for improving PA participation, but the Web-based intervention was more beneficial for increasing health education [36]. Moreover, Compernolle et al found that a tailored, Web-based PA intervention was most effective for participants who were insufficiently active at baseline [33]. Together, this evidence suggests that eHealth interventions have the potential to lead to effective health behavior change using tailored messages delivered through resource-efficient platforms. Although the USPTF review found improvements in some measures of cardiac health following clinician-based PA counseling [12], there continues to be a need for additional development and testing of feasible low-dosage PA interventions within health care delivery systems.

Exercise treadmill testing (ETT) is often one of the first diagnostic procedures performed on patients who present to their physicians with chest pain [37]. For those patients with positive test results, standard practice care follows, which includes consultations with cardiac specialists, intensive risk factor management, and careful clinical follow-up. For those patients with negative test results, there are no additional standard care services provided and often they are sent home with advice to monitor their dietary intake and exercise more. These patients may develop a perception of low risk of future cardiac events; however, these cardiac patients remain at a higher risk of mortality when compared to the general population [38]. A PA intervention delivered within the clinical setting may be an effective strategy for improving the health of this population at a time when they may be motivated to take preventive action.

The current study fills these gaps by testing the impact of a clinic-based, tailored, interactive computer session on PA participation using individual and environmental strategies. Most of the previous studies examined were not developed and integrated as part of the health care delivery system [12,30-32,39], particularly the ETT system, which presents an optimal opportunity for PA counseling for a high-risk population. This study used an integrated research-practice systems-based approach [40] and sought to incorporate the USPTF recommendation following negative ETT by developing and testing a low- to medium-intensity, theory-based, tailored PA intervention, delivered within the health care system.

The purpose of this study was to determine the effectiveness of a tailored, computer-based interactive personal action planning session to initiate PA among a group of sedentary patients. It was hypothesized that compared to controls, patients who completed the computer-based interactive personal action planning session, or received a voucher for a local fitness facility, or received both would significantly increase PA participation.

## Methods

### Design

This paper presents physical activity initiation information as part of a larger trial—Cardi*ACTION*, a 2x2 randomized controlled trial to test the efficacy of three PA interventions compared to a matched contact control group [41]. Participants were randomly assigned to one of four treatment arms: (1) increased environmental accessibility to PA resources (ENV), (2) a tailored, social cognitive PA intervention operationalized using the “5 As” (ask, advise, assess, assist, and arrange) (SC) [18,31,34], (3) combined PA intervention of both ENV and SC approaches (COMBO), or (4) a matched contact nutrition control (CON). For the larger trial, we powered our study to detect statistically significant changes in minutes of moderate to vigorous intensity PA at 6 months between conditions. For a more conservative estimate of the appropriate sample size, a Bonferroni correction was used by dividing alpha by the number of comparisons necessary with the 2x2 design. As such, to detect a 90-minute difference between conditions with a power of 95% and an alpha of .0083, a total of 93 participants per group were needed.

### Participants

Adults age 18 and over were recruited to the Cardi*ACTION* trial between April 2004 and April 2006 during an outpatient health care visit in which they completed an ETT. All patients were referred to a cardiac stress test by their primary care physician for diagnosis following symptoms related to cardiovascular disease (eg, heart palpitations, shortness of breath, chest pain) in an integrated health care system in the United States. The inclusion criteria for this study were (1) no chest pains and a normal electrocardiogram during the treadmill stress test and (2) currently physically inactive or insufficiently active (<150 moderate intensity PA/week). Patients were excluded from the study if they (1) were younger than 18 years of age, (2) had no access to a telephone, (3) were not able to read or understand English, (4) had a contraindication to PA identified during stress testing, and (5) already had a membership to a recreation center.

A total of 4097 patients completed ETT during the study recruitment period and 1300 participants met eligibility criteria (31.73%). The majority of patients were excluded because they reported sufficient PA (1507/4097, 36.78%), had a positive ETT (631/4097, 15.40%), had a gym membership (243/4097, 5.93%), were not able to read or understand English (138/4097, 3.37%), or had a contraindication to PA identified during stress testing (121/4097, 2.95%). Finally, a total of 157 patients were not approached for participation. Of those eligible who declined to participate (n=848), the most common reasons cited for declining included not being interested (n=314), being too busy (n=201), or not wanting to participate in research (n=119). A total of 452 patients (34.7% of eligible patients) completed a written informed consent, baseline assessments, and were enrolled in the study. Those eligible who enrolled in the study were more likely to be female (59.9% vs 47.0%) and African American (12.6% vs. 7.1%) when compared to those eligible who declined participation. There were no differences based on ethnicity, age, or PA between those who agreed and those who declined participation. Those enrolled were then randomly assigned to one of the four interventions: ENV, SC, COMBO, or CON. Group assignment was stratified based on gender (40% female) and abnormal heart rate recovery results (33% abnormal) following a randomization table created by the study statistician. Trained research assistants opened a pre-determined envelope with group assignment upon completion of all baseline measurements. To reduce potential bias, research assistants were blinded to the contents of the envelope.

### Procedures

All patients who met preliminary eligibility criteria were informed of the Cardi*ACTION* study by an exercise physiologist at the completion of the ETT. Those who showed interest in the study were referred to an onsite research assistant who explained the study in detail and conducted a short screening to determine eligibility. Patients eligible to participate in the study completed the informed consent process and a computer-based survey. A subsequent study visit at the treadmill center was then scheduled for 10 days after initial recruitment to complete the randomization process and initiate the study intervention. During the 10-day follow-up visit, all participants were randomized to one of the four interventions after completion of all baseline assessments and subsequently completed an interactive computer session specific to their intervention arm. All participants, regardless of condition, were matched on contact frequency and type. Specifically, following the computer interactive session, all participants received 3 interactive voice response (IVR) support calls and 3 tailored newsletters at 1, 3, and 5 months post randomization (see [41] for details). ENV group participants’ computer interactive session included an interactive geographic information system (GIS) interface that allowed participants to select a free 12-month voucher to a fitness facility in close proximity to their home or workplace or the path in between home and work. SC group participants’ session included personal action planning to improve self- and response-efficacy as well as personal goals for PA. COMBO group participants’ session included both the ENV and SC components. CON group participants’ session targeted goals and personal action planning strategies for healthy eating.

The interactive computer session, its components, development and theoretical underpinnings, and all subsequent intervention components have been described elsewhere [41]. Briefly, the interactive computer session allowed the use of audio information, visual graphics, text, video, and the use of familiar models and locations. Videos for all four arms took approximately 20 minutes to complete and were filmed in the clinic’s treadmill center and featured the clinic’s Chief of Cardiology. The SC, ENV, and COMBO interventions began with an opening message about the importance of PA to achieving good health, while the CON intervention focused on the importance of healthy eating.

Following the opening message, participants in the SC and COMBO group proceeded through an interactive action planning session based on the 5 As [42]. The session included an *assessment* of the patient’s PA level and motives for increasing PA and provided *advice* on the recommended level of PA. The interactive computer program then provided a range of minutes of PA that would be an appropriate starting point for the patient and used a collaborative goal setting process to *agree* on a goal for the upcoming month. Finally, the interactive session included *assisting* the patients with PA barrier identification and strategies to overcome barriers, followed by *arranging* for follow-up with an automated telephone counseling call 1 month later [42]. The CON group followed the same process with a focus on healthy eating (ie, personal action plan to increase fruit and vegetable consumption and decrease fat intake). The ENV group participants proceeded through a mapping tool (GIS) designed to help them identify fitness facilities close to their homes and/or work. Participants were able to look at facilities by location and amenities available. At the end of the session, participants in the ENV group printed their free 12-month membership voucher to their facility of choice. The COMBO group completed the GIS mapping tool after completing a personal action plan using the 5 As, as described above.

One month after the completion of the interactive computer session, all participants received a follow-up interactive voice response (IVR) automated telephone call. The IVR system pulled individual information (ie, PA and eating behavior, goals, strategies, and barriers selected) directly from the CD-ROM session and called participants at designated times on multiple days over a week to increase the likelihood of participant completion. All participants completed a PA assessment as part of the IVR follow-up. The study design and protocol was approved by the Kaiser Permanente Colorado Institutional Review Board and is registered (NCT00432133).

### Measures

Physical activity was defined as the total minutes of moderate and vigorous PA performed each week, measured using PA participation questions from the Centers for Disease Control and Prevention Behavioral Risk Factor Surveillance Survey [43]. This measurement tool has shown validity and reliability in determining respondent PA levels [44,45]. This tool requires participants to respond to a series of questions regarding PA intensity (light, moderate, and vigorous), frequency (number of days per week), and duration (number of minutes per day) in a typical week. The PA measure was integrated into the computer interactive session as well as the IVR follow-up call. These responses were used to calculate total weekly minutes of moderate and vigorous PA and the percentage of study participants meeting current recommendations for 150 minutes or more of moderate and vigorous PA per week.

### Statistical Analysis

Statistical analysis (IBM SPSS v21.0) included descriptive statistics of sex, age, race, ethnicity, and baseline minutes of moderate and vigorous physical activity. Chi-square and independent *t* tests were conducted to determine if any of the groups differed on baseline characteristics (Table 1). A general linear model for repeated measures analysis was conducted using a within-subjects factor of time (baseline and 1-month follow-up), between-subjects factor of group (ENV, SC, COMBO, and CON), and change in weekly PA minutes between baseline and 1-month as the primary dependent variable. Post hoc analyses were conducted to investigate any significant effects found with alpha set at .05.

Additionally, the percentage of all participants currently meeting public health recommendations for aerobic exercise at 1-month follow-up was calculated by group. A dichotomous variable was developed to indicate whether participants were meeting public health recommendations of 150 minutes moderate to vigorous PA minutes per week. Logistic regression models were conducted to determine if study group had a significant impact on attainment of PA public health recommendations.

Finally, we used intention-to-treat (ITT) analysis to include all participants with non-missing baseline outcome measurements (7 participants had missing baseline values). For those participants with missing 1-month follow-up PA measurement (37/452, 8.18%), we replaced the missing 1-month data with their baseline value following the Last Observation Carried Forward approach. We also conducted subgroup analysis including only those participants who cashed their vouchers in the COMBO (67/125, 53.6%) and ENV (63/115, 54.78%) groups, and our results remained unchanged. As such, we present the results of our final ITT analysis with 445 participants.

## Results

### Participant Characteristics

**Figure 1:**
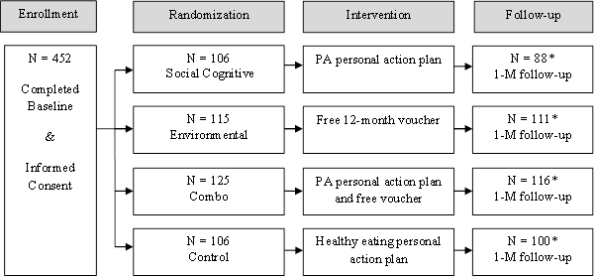
Flow diagram of participants’ progress through study.

Figure 1 depicts the flow diagram of participants through the different stages of the study. In total, 452 cardiac patients were enrolled in the study and completed baseline assessments and the interactive computer session. The average age of the participants was 58.6 years (SD 9.6) and 59.9% were women. Over three-quarters of the sample was white and 12% and 10% of the sample were African American and Latino, respectively. At 1-month follow-up, 91.8% (415/452) of participants completed the automated phone calls and provided data included in the analysis. Although the remaining 8.2% (37/452) of participants did not complete the 1-month follow-up IVR call, no participants had actively withdrawn from the study. Participants who were randomized to the SC group (*F*_1,3_=5.254, *P*<.001; SC=83%, ENV=96.5%, COMBO=92.8%, CON=94.3%) were significantly less likely to complete the 1-month IVR follow-up than their counterparts. No significant differences in non-completion were found among the other groups. Furthermore, there were no significant differences in 1-month IVR call completion by sex, education, race, ethnicity, marital status, income, employment, age, and weekly PA participation, both within and between groups. Baseline characteristics for all study participants are shown on Table 1. Baseline measurements did not differ significantly between the four intervention groups.

**Table 1:** Participant characteristics at baseline.^a^

Participant characteristics	Environmental (n=115)	Social cognitive (n=106)	Combination (n=125)	Control (n=106)	Overall (n=452)
Age, mean (SD)	58.77 (9.49)	59.62 (9.48)	58.06 (10.12)	57.92 (9.55)	58.57 (9.67)
Female, %	53.9	61.3	60	62.3	59.3
Black, %	13.2	9.5	12.9	11.3	11.8
Hispanic or Latino, %	9.6	11.4	12.1	12.3	11.4
High school or less, %	36	39	44.4	40.6	40.1
Married, %	69.3	60	69.4	67	66.6
USD $30,000 or less, %	10.5	14.3	8.9	15.1	12
Employed full-time, %	66.7	67.6	67.7	64.2	66.6
Moderate and vigorous minutes of PA, mean (SD)	65.77 (65.3)	63.38 (78.2)	56.25 (66.72)	80.50 (89.1)	65.9 (75.1)

^a^No significant differences between groups.

### Changes in Physical Activity: Weekly Minutes of Moderate and Vigorous Physical Activity

Table 2 shows that participants from three study groups (SC, ENV, and COMBO) increased their level of PA at 1 month follow-up, while participants from the CON group reported a small decrease. A repeated-measures analysis with all participants found a main effect for time indicating that PA increased across the study sample from baseline to follow-up (*F*_1,441_=30.03, *P<*.001). A time by condition interaction (*F*_3,441_=8.33, *P<*.001) followed by post hoc analyses indicated that SC participants exhibited a significant increase in weekly PA participation (mean 45.1, SD 10.2) compared to CON (mean -2.5, SD 10.8, *P*=.004), and ENV (mean 8.3, SD 8.1, *P*<.05). Additionally, COMBO participants exhibited a significant increase in weekly PA participation (mean 53.4, SD 8.9) compared to CON (mean -2.5, SD 10.8, *P*<.001), and ENV (mean 8.3, SD 8.1, *P*=.003) participants. There were no differences between ENV and CON or between SC and COMBO.

**Table 2:** Study outcomes by condition and time.

Weekly minutes of moderate and vigorous PA	Environmental (n=113)	Social cognitive (n=105)	Combination (n=124)	Control (n=103)	Overall (n=445)
Baseline, mean (SE)	65.76 (7.04)	63.38 (7.30)	56.25 (6.72)	80.49 (7.37)	66.47 (3.55)
1 month, mean (SE)	74.04 (8.37)	108.46 (8.69)	109.71 (7.99)	77.97 (8.77)	92.55 (4.23)
Difference	+8.28^a^	+45.08^b^	+53.46^c^	-2.52^a,b,c^	+26.07
Meet PA public health recommendations, 1 month, %	15.9	27.6^d^	29.0^d^	15.5	22.2

^a^Denotes no difference between ENV and CON groups.

^b^Denotes difference between SC and CON groups: *P*=.004.

^c^Denotes difference between COMBO and CON groups: *P*<.001.

^d^Significantly different from control group, χ^2^_3_=10.343, *P*=.016.

### Changes in Physical Activity: Meeting Physical Activity Public Health Recommendations

Table 2 depicts the percentage of participants in each study group who achieved public health recommendations. Since no participants met public health recommendations at baseline, these percentages represent total increase from baseline to 1-month follow-up. At 1-month follow-up, 15.9%, 27.6%, 29.0%, and 15.5% of the participants for the ENV, SC, COMBO, and CON groups, respectively, met the PA recommendations for aerobic activities. Chi-square test results indicate that SC and COMBO groups had significantly higher prevalence of meeting recommendations than CON group, χ^2^_3_=10.343, *P=*.016, while ENV participants did not significantly differ from CON participants.

Binary logistical regression analysis with all participants showed that SC (*P*=.036) and COMBO (*P*=.017) participants were significantly more likely to meet public health PA recommendations when compared to CON participants. ENV participants showed no significant difference from CON participants on meeting PA recommendations. Regression coefficients are shown in Table 3. Odds ratio results suggest that participants in the SC group (OR 2.075, 95% CI 1.047-4.110) and COMBO group (OR 2.224, 95% CI 1.151-4.301) were 108% and 122%, respectively, more likely to achieve current public health recommendations for aerobic exercise than their CON group counterparts. There were no differences between ENV group participants and CON group.

**Table 3:** Likelihood of meeting PA recommendation results (control group set as reference category).

Study group	*B*	SE	Wald	Sig.	OR	95% CI for exp. B
Environmental (n=113)	.030	0.374	0.006	0.937	1.030	0.495-2.145
Social cognitive (n=105)	.730	0.349	4.380	0.036	2.075	1.047-4.110
Combination (n=124)	.800	0.336	5.650	0.017	2.224	1.151-4.301

## Discussion

### Principal Findings

This study sought to determine whether a brief, tailored, computer-based, interactive personal action planning session operationalized using the 5 As could initiate increased PA participation among a group of sedentary cardiac patients. We hypothesized that participants who completed the computer-based interactive personal action planning session would exhibit significant increases in PA participation compared to control participants. This hypothesis was supported. We found that participants assigned to the SC and COMBO study arms experienced significant increases in PA at 1 month compared to CON participants who decreased their PA participation. This is a particularly important finding given that the current evidence on the effectiveness of tailored, technology-based PA programs is mixed at best [12,30-32]. In fact, Becker et al suggested that it would be unreasonable to expect PA change from low-dosage interventions [46]. Our findings suggest the contrary, showing that a brief, low-dosage, tailored, computer-based interactive personal action planning session can in fact lead to PA behavior initiation among previously sedentary adults.

An ancillary aim of this study was to determine whether environmental or social-cognitive mechanisms to PA behavior change may impact PA initiation differentially. We found that participants who were exposed to the social-cognitive approach (SC and COMBO) exhibited significantly greater PA increases at 1 month compared to participants in the control group. On the other hand, participants who were exposed only to the environmental condition (ENV) showed no differences from the control condition. Together these findings suggest that a social cognitive approach to initiating PA may be a superior approach than only providing environmental access to PA through a complementary fitness facility membership.

Nevertheless, even though SC participants experienced the greatest increase in PA during the study period, participants in this arm also had a higher rate of non-completion of IVR calls. This was an unexpected but interesting finding. One explanation may be that non-completion was higher for SC participants due to the demand of the SC intervention relative to the ENV intervention. On the other hand, participants in the COMBO arm had a lower rate of non-completion even though they were also exposed to the SC content. Because the only intervention difference between the SC and COMBO arms was that the latter was exposed to the ENV content, an alternative explanation may be that the fitness facility access provided an incentive for participants to remain in the study and therefore increased the completion rate. These findings appear to suggest that while an individual approach may have a greater impact on PA initiation, environmental factors may also play a key role in the ongoing engagement of participants. As such, the combination of the environmental intervention with the personal action planning approach appears to lead to both increased rates of PA participation and better IVR completion rates overall.

### Strengths and Limitations

This study was not without limitations. First, PA was measured using self-reported minutes of PA, leaving room for reporting error and/or bias. However, we suspect that participants may have provided more honest estimates of their PA participation since they were simply typing these numbers into the telephone keypad rather than reporting them directly to a research staff member, potentially reducing response bias [47,48]. In particular, self-administered questionnaires are thought to be completed in a more relaxed and honest manner without the pressure of an external interviewer [47]. Our IVR approach may have provided participants with a more relaxed experience without the presence of the interviewer and with the convenience of completing the questionnaire on their own time at a location of their choosing (participants were allowed to select dates and times for IVR completion that were the most convenient for them). Nevertheless, the effect sizes observed in the current study must be interpreted with caution given the inherent danger of overestimation of physical activity behavior when using self-report measures. Second, change in PA was reported over a relatively short timeframe of 1 month. Although we observed significant increases in PA at 1 month, it is unclear whether this increase in PA participation would be maintained over a longer period of time. Future studies should examine the impact of an interactive computer intervention on long-term PA participation.

One of the strengths of this study was the low resources-high reach mode of intervention delivery. The interactive computer approach allowed for a tailored intervention that required minimal resources for delivery within a health care setting. Furthermore, the IVR contact at 1 month was tailored based on the participant’s responses to the baseline interactive computer session and allowed for collection of additional data all while using minimal resources. Results from this study support the concept of a minimum intervention needed for change [49]. The interactive computer intervention used in this study was resource efficient and resulted in significant behavior change over a short period. As such, because of the feasibility of our interactive computer intervention delivered within the ETT system, this approach has potential for rapid and broad adoption and offers promise for integration within health care settings.

### Conclusions

In general, our findings indicate that a social cognitive approach to PA behavior change may lead to a greater increase in PA than simply providing access to a fitness facility. However, this approach alone seemed to lead to higher IVR non-completion rates. While a single exposure to an interactive computer session was sufficient to lead to PA initiation, this approach together with providing access to a fitness facility appeared to be the best combination in order to initiate PA and increase IVR completion rates. These findings provide initial evidence that brief, low-dosage, tailored, interactive technology-based interventions to initiate PA can be feasibly delivered within the health care setting, in particular as part of the ETT system, and lead to increased PA among sedentary cardiac patients.

## Abbreviations

5 As: Ask, Advise, Assess, Assist, and Arrange
AHA: American Heart Association
CHD: coronary heart disease
COMBO: combined PA intervention arm of both ENV and SC approaches
CON: matched contact nutrition control arm
ENV: increased environmental accessibility to PA resources arm
ETT: exercise treadmill testing
GIS: geographic information system
IVR: interactive voice response system
PA: physical activity
SC: tailored social cognitive PA intervention arm
USPTF: US Preventive Task Force
